# Assessment of Unusual Gigantic Jets observed during the Monsoon season: First observations from Indian Subcontinent

**DOI:** 10.1038/s41598-017-16696-5

**Published:** 2017-11-27

**Authors:** Rajesh Singh, Ajeet K. Maurya, Olivier Chanrion, Torsten Neubert, Steven A. Cummer, Janusz Mlynarczyk, Morris B. Cohen, Devendraa Siingh, Sushil Kumar

**Affiliations:** 1 KSK Geomagnetic Research laboratory, Indian Institute of Geomagnetism, Allahabad, 221505 India; 2 Atmospheric Physics Lab, Department of Physics, Banaras Hindu University, Varanasi, 221005 India; 3 National Space Institute, Technical University of Denmark (DTU Space), Elektrovej 327, 2800 Kgs. Lyngby, Denmark; 4 Electrical and Computer Engineering Department, Duke University, Durham, USA; 5 AGH University of Science and Technology, Department of Electronics, Krakow, Poland; 6 Electrical and Computer Engineering, Georgia Institute of Technology, Atlanta, USA; 7 Indian Institute of Tropical Meteorology, Pune, India; 8 School of Engineering and Physics, The University of the South Pacific, Suva, Fiji

## Abstract

Gigantic Jets are electric discharges from thunderstorm cloud tops to the bottom of ionosphere at ~90 km altitude and electrically connect the troposphere and lower ionosphere. Since their first report in 2002, sporadic observations have been reported from ground and space based observations. Here we report first observations of Gigantic Jets in Indian subcontinent over the Indo-Gangetic plains during the monsoon season. Two storms each produced two jets with characteristics not documented so far. Jets propagated ~37 km up remarkably in ~5 ms with velocity of ~7.4 × 10^6^ms^−1^ and disappeared within ~40–80 ms, which is faster compared to jets reported earlier. The electromagnetic signatures show that they are of negative polarity, transporting net negative charge of ~17–23 C to the lower ionosphere. One jet had an unusual form observed for the first time, which emerged from the leading edge of a slowly drifting complex convective cloud close to the highest regions at ~17 km altitude. A horizontal displacement of ~10 km developed at ~50 km altitude before connecting to the lower ionosphere. Modeling of these Gigantic jets suggests that Gigantic Jets may bend when initiated at the edge of clouds with misaligned vertical charge distribution.

## Introduction

The Gigantic Jets (GJs) are the most spectacular of the family of discharges above thunderstorms, which includes the Red Sprites of the mesosphere and the Blue Jets from clouds into the stratosphere^1–4^. GJs were first reported in 2002 over the Caribbean Ocean in light-sensitive video taken from the Arecibo observatory site on Puerto Rico and have since been observed mostly from a satellite at large distances^5–8^, with only a few tens of events from the ground at higher resolution^9–13^. GJs are not well understood because of the sparsity of observations and the complexities of their nature. They span more than four orders of magnitude of the neutral atmospheric density in which they propagate, from a number density of ~10^23^ m^−3^ at cloud tops to ~10^19^ m^−3^ at 85 km altitude. Studies suggest that they are leaders at the lower altitudes within the stratosphere and streamers at the higher altitudes within the mesosphere^14^. Leaders and streamers are two principal modes of electric discharges and are important for lightning propagation, however, their interaction is poorly understood because of the challenging temporal scales (ps) at ground pressure. This is because the time-scales are reversely proportional to the pressure and therefore shorter at ground pressure. In the mesosphere, both temporal and spatial scales are 4–5 orders of magnitude larger^15^, bringing them into reach of camera imaging capabilities. GJs, therefore, are not only interesting in their own right, but also offer an opportunity to study the electric discharges in the natural laboratory of the tenuous upper atmosphere, with implications for lightning dynamics and technological applications.

It has been proposed that the electric fields from charge imbalances in the upper half of a thundercloud stimulate the discharges, either from fields between charge regions of opposite polarity within the cloud (Gigantic Jets) or between an upper charge region and the screening layer at the cloud top (Blue Jets)^16,17^. The meteorological conditions during GJ events appear to vary depending on source location. GJs reported over North America point to tropical-like environment with high cloud tops reaching above 13 km and convective surges^18,19^. In contrast, a GJ over the Mediterranean Sea during the winter appeared from clouds reaching only ~6.5 km altitude^10^. Here we add to these studies by analyzing four GJs observed during 2013 and 2014 for the first time over the Indian mainland in the Indo-Gangetic plain. The jets were observed during two thunderstorms with cloud altitude reaching ~17 km with a short life span of ~40–80 ms and one of them was peculiarly horizontally tilted.

A camera system for observations of Sprites, Blue Jets and GJs above thunderstorms, known collectively as the Transient Luminous Emissions (TLEs), was deployed by the Indian Institute of Geomagnetism (IIG) in 2012^20^. The site is outside the city of Allahabad at 25.4°N, 81.9°E, a location from which the cameras cover thunderstorm activity in the middle of Indo-Gangetic plain. The system has two light-sensitive Watec 902 H cameras, one with a 16 mm, f1.4 lens with a horizontal field of view (FOV) 26.5° and vertical FOV 15°, the other with a 50 mm, f0.95 lens with horizontal FOV 7.25° and vertical FOV 4.8°. The cameras are monochrome of sensitivity above 0.002 lux and run at 25 frames per second. Both cameras are co-aligned on a common platform mounted in a weather proof housing on top of a QuickSet20 motorized pan-tilt unit, which allows for pointing within 360° azimuth (pan) and −35° to + 35° elevation (tilt). The system includes a control computer for remote control via the Internet. The computer runs trigger software that captures optical event images and time-stamps the images using GPS receiver^21^.

**Figure 1:**
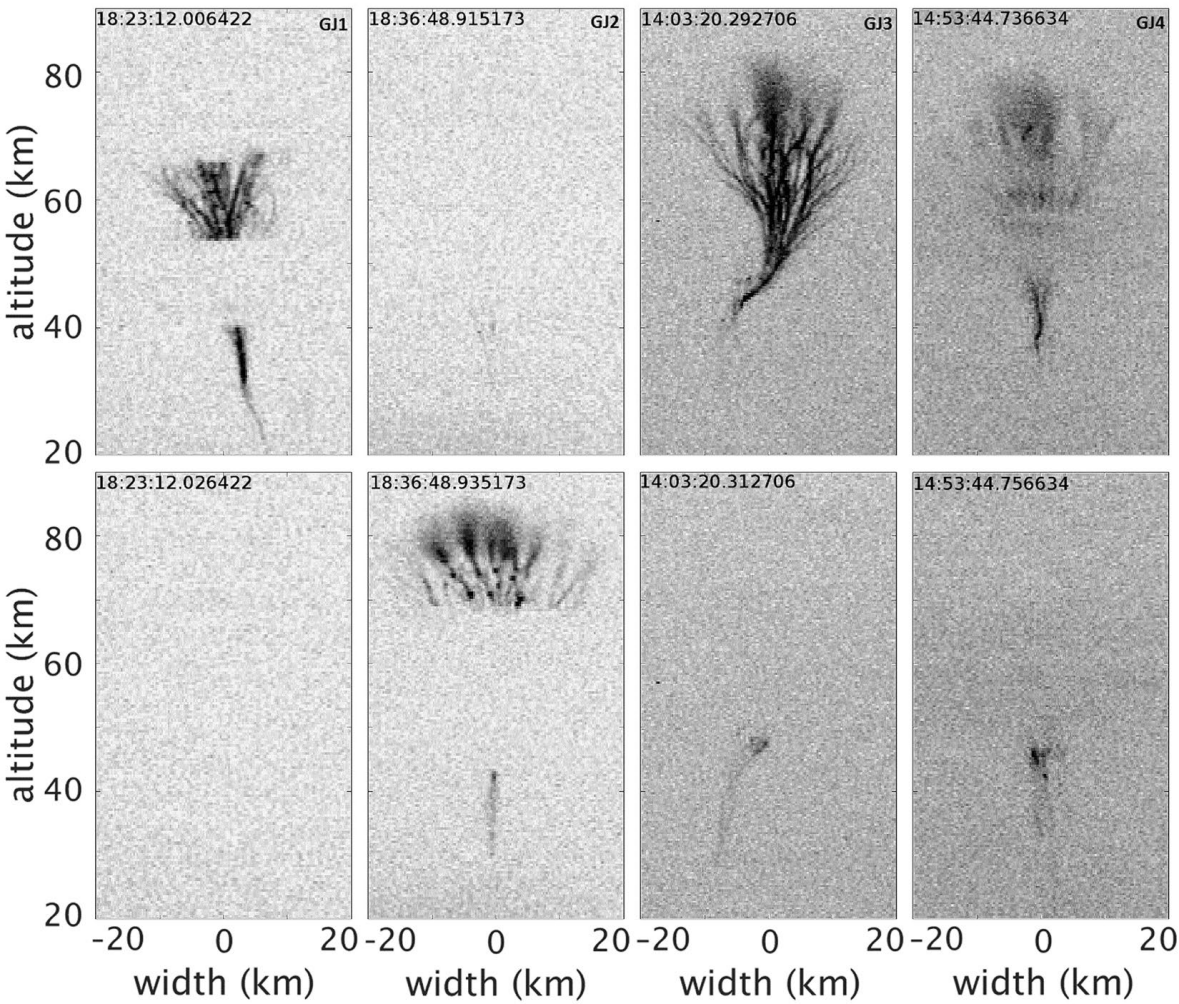
Gigantic Jets records on 02 August 2013 (GJ1, GJ2) and 07 September 2014 (GJ3, GJ4) at a site (25.4°N, 81.9°E**)** near Allahabad, India. The grayscale is inverted, bright appearing dark. The altitude is estimated with an error of +/−1.2 km derived from an error estimate of GJ position of +/−5 km.

The four GJs observed from our site are shown in Fig. 1. The two in the left side columns (GJ1 and GJ2) were recorded on 02 August 2013 at 18:23:12.02 UT and 18:36:48.94 UT, respectively, at a distance ~430 km southwest of Allahabad. GJ1 appeared only in one image, apparently fully developed and extinguished within the 40 ms exposure time. GJ2 appeared fully developed in the second frame with the lower portion in the first frame. The bottom part of GJ2 is partly obscured up to ~20 km altitude by haze and the middle portion of GJ1 and GJ2 were obscured by clouds between the jets and the recording system. We did not record any other TLE on this day. The two jets (GJ3 and GJ4) in the right side columns were captured on 07 September 2014 at 14:03:20.31 UT and 14:53:44.76 UT, respectively, at a distance of ~350 km south of Allahabad. These events lasted two frames each, both fully developed during the first frame with the lower portion, the “stem”, lasting a second frame. Also for these jets, the bottom parts are obscured in a haze. Apart from GJ3 and GJ4 we also recorded two sprites at 14:05:37 UT and 14:05:54 UT respectively. The structure of GJ3 is remarkable, bending ~10 km towards the horizontal direction at ~50 km altitude before branching upwards. Common features of all jets (GJ1-GJ4) reported here are their fast rise times and short lifetimes ~40–80 ms, which are short compared to most reported GJs (life time ~400–700 ms) previously from other locations around the globe^5,8,9^. The short duration of maximum 80ms is estimated despite cloud obscuration of GJ1–2, assuming their middle part unlikely re-brightens after full development.

**Figure 2:**
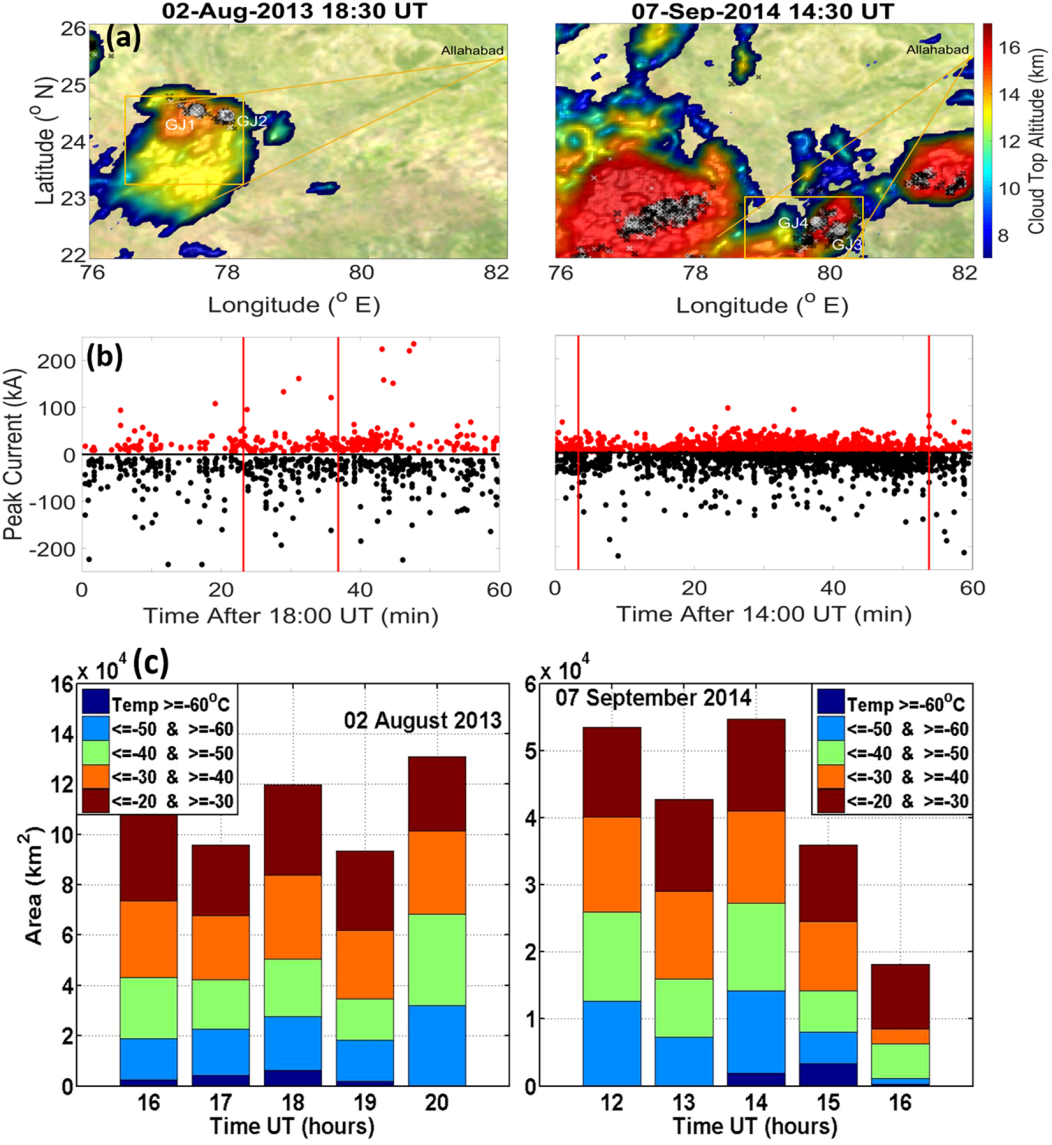
(**a**) Storm and lightning activity for GJ1, GJ2 on August 2, 2013 (left panels) and for GJ3, GJ4 on September 7, 2014 (right panels). Top: The cloud top altitude estimated from the Meteosat satellite with the location of the jets and cloud-to-ground (CG) lightning during a 5-min period around each jet. Positive CGs are marked with “+” and negative CGs with “−”. Also shown is the location of Allahabad station and the field of view of the wide-field camera. The maps are plotted using the python library VisVis 1.8 (https://pypi.python.org/pypi/visvis/1.8) on top of a Visible Earth NASA image available at http://visibleearth.nasa.gov/view.php?id=57730. The resulting images are encapsulated in a Matlab R2015b figure (https://se.mathworks.com/products/matlab.html). (**b**) The peak current of CG lightning for one hour and in a 1° × 1° region around the time and location of the jets is shown. (**c**) Evolution of cloud top temperature derived from MATSAT-1 satellite for the area shown as boxes in panels (**a**) (http://weather.is.kochi-u.ac.jp/archive-e.html).

The distance of the jets from the cameras, which is required to estimate their altitude, is estimated from the location of lightning activity nearest in time. This fits very well with the viewing angles of the jets. Figure 2a (top panels) shows lightning activity detected by the global lightning detection (GLD) 360 network^22,23^ in a 10 minute period around the occurrence time of jet events. They are superimposed on cloud altitude maps derived from cloud images from the Meteosat satellite taken around the time of the events at 18:30 UT (2013) and 14:30 UT (2014). Also shown are the estimates of jets locations, the camera location and FOV of the wide-angle camera lens. The estimated generation altitude of ~17 km and the termination altitude of ~70–90 km are consistent with past observations^5,8^. It is still to be noted that the murky atmosphere made the observation of weak part of the jet difficult and can explain why the termination altitude seen in Fig. 1 appears lower than in other works.

The peak current of cloud-to-ground (CG) as detected by GLD360 network during one hour and in a 1° × 1° region around the time and location of the jets is shown in the panel Fig. 2b. The 2013 storm generated 262 recorded CGs, with an average lightning rate of ~4.5 CG min^−1^, which is relatively low compared to typical rates of ~3.7 to 14 per minute^21,22^. ~75% of these were with negative polarity. The 2014 storm with an average lightning rate of ~14 CGs/min generated 837 CGs with ~68% of negative polarity. The maximum peak current for positive and negative CGs was 252 kA and −280 kA in 2013 storm and 203 kA and −206 kA in 2014 storm, suggesting that the storm in 2013, though less electrically active, had stronger electric charge regions. As seen in Fig. 2b, the lightning activity is fairly constant throughout the 1-hour period with no apparent change around the time and location of the jets occurrence. Which suggests a mesoscale convective system (MCS), a complex of thunderstorms organized during post monsoon break period. To further understand the MCS we also analyzed the meteorological parameters from a skewT-logP diagram from the nearest available balloon sounding station of Bhuvnewsher (India) on August 02, 2013 at 1200 UT and September 07, 2014 at 0000 UT. Although the skewT-logP diagram is not shown here, interested readers can find it at http://weather.uwyo.edu/upperair/seasia.html. The wind is from the South until 6500 m and 7500 m respectively for both GJ events, then it turns and comes from the East above that. The wind become quite strong above ~12000 m reaching speeds of ~30 knots for both the events. Further, the overall wind speed is higher for 2014 event compared to 2013 event. The CAPE for 2013 and 2014 events is 2471 J/kg and 1164 J/kg respectively, suggesting more turbulence and deeper convection for the August 02, 2013 MCS thunderstorm. Figure 2c, shows the cloud top temperature area evolution within the boxes shown in Fig. 2a during 16–20 UT and 12–16 UT for the GJ events of August 02, 2013 and September 07, 2014 respectively. For this purpose, we have used MTSAT-1 cloud top temperature derived from the IR1 (10.3–11.3 μm) band observations. The data is available hourly from [http://weather.is.kochi-u.ac.jp/archive-e.html]. As seen from Fig. 2(c) for 02 August 2013 GJs at ~18:30 hrs, the coldest temperature (> = −60 C) region was maximum during GJ occurrence hour (18 UT), and further, areas with other temperature range was also largest during this time (18 UT). In case of 2014 event, the coldest temperature (> = −60 C) region continued to evolve before and after the GJ occurrence hour (14 UT). But areas with other temperature range was largest during this hour (14 UT), similar as for 2013 GJ events. Cloud top evolution suggests deep MCS was prevalent before and after the time of both observed GJs.

**Figure 3:**
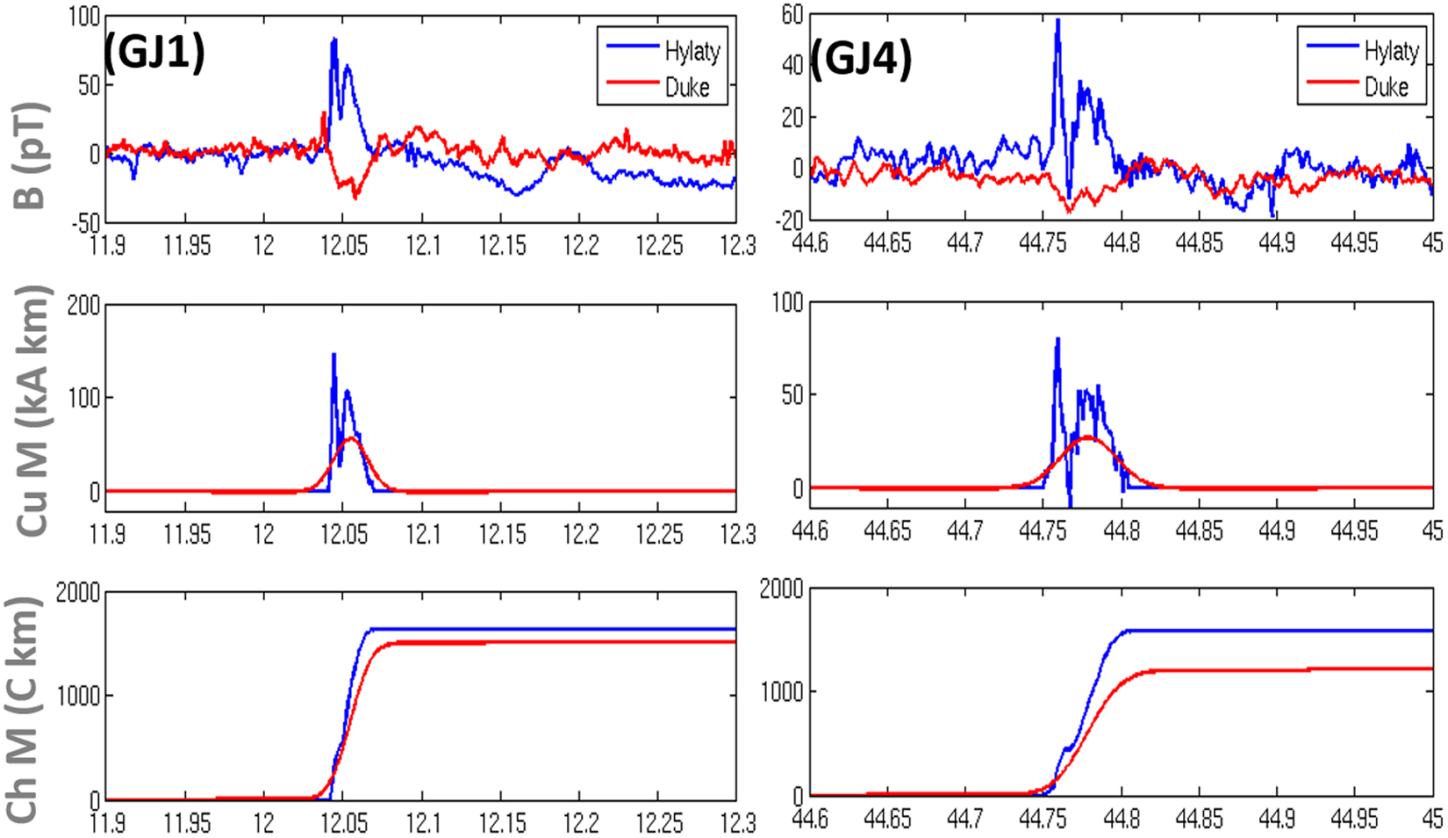
ULF magnetic data as functions of time for GJ1 (left) and GJ4 (right). The blue curves correspond to observations from the site Hylaty and the red curves from Duke. Time is relative to start time of first GJ image. The ULF data are time shifted to the source location. The top panel is the magnetic field variation associated with GJs, middle and bottom panels are corresponding current and charge moment change associated with GJs.

The current moment waveform and charge moment change of the jets were determined from ULF/ELF electromagnetic wave measurements at two sites. One is Hylaty (49.20°N, 22.54°E) in the Bieszczady Mountains in Poland^24^, which continuously measures two magnetic field components in a frequency band from 0.03 Hz-300 Hz. The second site (35.97°N, 79.10°W) is at Duke University in the USA which measures two magnetic field components from 0.01–400 Hz^9^. The data from both locations allowed estimation of charge and current moments for GJ1 and GJ4. We were not able to associate GJ2 and GJ3 with the corresponding ELF signals reliably enough to calculate the charge moment change. Bottom three panels of Fig. 3 show the azimuthal magnetic field component, the current moment and the charge moment for GJ1 and GJ4. Blue curves correspond to observations from the site Hylaty and the red curves from Duke. The data are shifted in time corresponding to the delay of signal propagation from the events location to the receivers and the delay introduced by the receivers. The distance to the receiver in Poland is ~5,500 km corresponding to ~18 ms delay and to Duke ~13,000 km with ~43 ms delay. Although the signal is weak at Duke, it is possible to identify the direction of arrival of the electromagnetic signal at both stations for both jets, which are consistent with the GJ locations. Additionally, the round-the-world wave is clearly visible in the Hylaty recording of GJ1. Its presence allowed us to estimate the distance from the source and the obtained location is consistent with the GJ locations in the camera observations. Both stations conclude that the GJs are negative, transporting net negative charge to ionosphere.

The signal amplitude and the derived current moments of the Hylaty observations are double peaked for both GJs which is very unique and not seen in any GJ report before. The first peak can be interpreted as the establishment of the lower portion of the jet to ~50 km, a leader “stem”, and the second peak can be related to a streamer-type flash from the tip of the stem to the ionosphere, creating electrical connection between the cloud and the lower ionosphere^12^. In the case of GJ1, the lower portion of the jet propagated from the cloud top to ~50 km i.e. ~37 km in a duration of ~5 ms estimated from the rise time of the current moment found in Fig. 2, that is, with an average velocity ~7.4 × 10^6^ms^−1^ ^5,8^, during leader phase which is fast compared to reported jets^25,26^. It is of the order of streamer velocities and larger than typical leader velocities. After the first peak, there is a drop followed by a second smaller peak which may correspond to an in-cloud or GJ process^6^, or an equivalent to the return current in usual lightning^27^. Note, that while the optical images of GJ1 and GJ4 suggest jet activity lasting <40 ms and <80 ms, which is consistent with the main peaks of the current moment lasting ~40 ms for both events, both events have extended tails in the ULF measurements and return to their background value only after 80 ms (GJ1) and 120 ms (GJ4). The current moment peak values are estimated to 150 and 56 kAkm for GJ1 and 80 and 27 kAkm for GJ4, with the lower numbers for the Duke data. The values of Hylaty are consistent with past reports^12^ and are probably more reliable because the station is closer to the source. The values of charge moment change are consistent between the two stations. For GJ1, it is estimated to 1600 and 1500 Ckm and for GJ4 at 1560 and 1200 Ckm. It should be noted that the Hylaty and Duke teams use different propagation models and algorithms to estimate current and charge moment change^9,28^.

**Figure 4:**
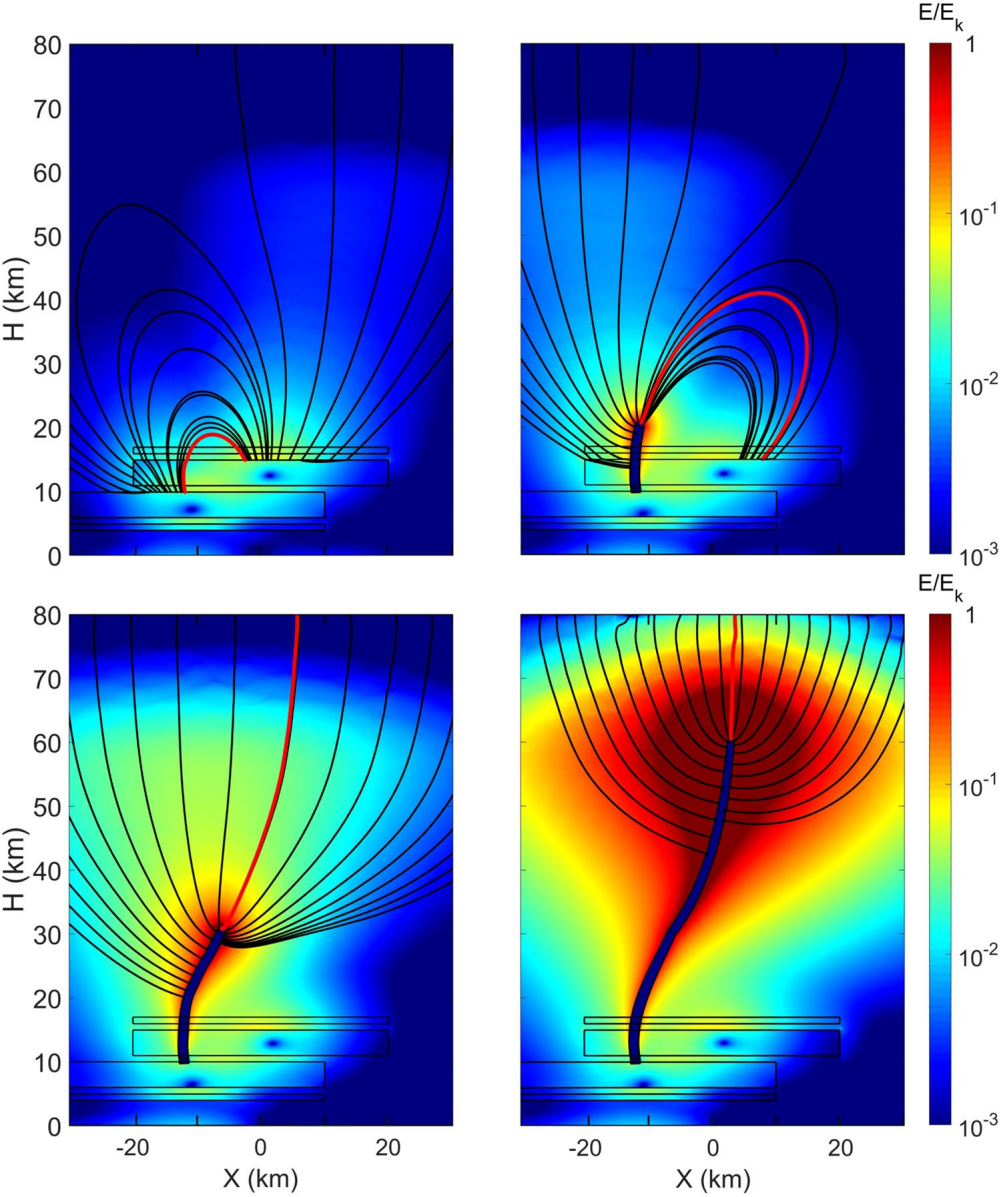
The evolution of the field structure above a vertically misaligned charge structure and a leader initiated from the main negative layer. The electric field (E) lines are shown in black, the jet is following the one in red. Details on the charge layers composition are given in the text. From the upper-left to the bottom right, the panels represent the field when the jet has respectively started, reached 20, 30 and 60 km altitude.

One of the jets (GJ3 of Fig. 1) appears markedly crooked, the lower portion being bent towards the horizontal direction, before flashing to the lower ionosphere. The structure of the jets can be understood by considering the electric field above a thunderstorm cloud. GJs of negative polarity, discharge a negative charge layer of a cloud to the ionosphere. It has been proposed that breakdown is triggered by a lesser charged positive layer above and that the discharge continues upwards towards the ionosphere driven by the electric field resulting from the charge distribution inside the cloud^16,17,29^. In Fig. 4, we show the evolution of the field structure above such cloud in which we have vertically misaligned the bottom and top charge layers and propagated a leader initiated from the main negative layer. The model of the cloud charge structure is built from four regions of electrically charged layers with spatial dimension and charge content matching a cloud producing giant jets [Fig. 3 of^16^]. The charge layers are cylindrical with 20 km radius, the main central charge layers are 4 km height and the top and bottom screening layers are 1 km height. From the top to the bottom, the charge layer centers are positioned at 16.5, 13, 8 and 4.5 km altitude and they contain a charge of −3, 82, 5, −120 and 25 C, respectively. In all layers, the charge is distributed horizontally as a Gaussian distribution of size 5 km and is distributed vertically uniformly. To misalign the charges vertically, the two top layers are horizontally shifted by 10 km from the bottom ones. The ground and ionosphere potential is at 0 V and 400 kV, respectively, and the atmospheric conductivity profile is assumed stationary and obtained from [Fig. 2 of ^30^]. The simplified model of the jet is built from a step by step propagation of a leader represented by a tube of radial dimension 750 m along field lines calculated iteratively. Each step is a few kilometers long, completed by half a sphere forming a shape that is assumed perfectly conductive. The first step is initiated at the top of the main negative charge layer from which we set the potential of −200 MV on the boundary of the tubular conductive path representing the jet. Assuming the leader follows the field line of highest field magnitude, successive steps are following the one initiated at the top of the half-sphere of the preceding step and share the same potential. The process is iterated until the jet had reached 60 km altitude.

The plot on Fig. 4 is given in the same viewing geometry as GJ3 of Fig. 1. It shows in color the magnitude of the reduced electric field E/E_k_, where E_k_ is the breakdown electric field, and the electric field (E) lines are shown in black, the jet is following the field line shown in red. From the upper-left to the bottom right, the panels represent the field when the jet has respectively started, reached 20, 30 and 60 km altitude. Following the propagation from the edge of the layer we see that the jet first curves horizontally toward the cloud top to a distance ~10 km horizontally, before it, at around 45 km altitude, curves back as it reaches towards the ionosphere. That same topology and magnitude of curvature is seen in GJ3. Since the model is simplified and assume a configuration among many, further work is needed to understand how a jet propagates to ~50 km altitude, flashes to the ionosphere, and remains ignited in the section below ~50 km. Nevertheless, Fig. 4 gives a framework for understanding the overall structure of jets and hints that GJs may bend when initiated at the edge of giant jet producing clouds with misaligned vertical charge distribution. Still, we cannot exclude alternative explanations of the abruptness of the curve which could come from slightly charged layers in the middle atmosphere, e.g. from volcanic aerosols or meteoric dust particles.

Gigantic Jets propagate in a combination of leaders and streamers and electrically connect the troposphere and the lower ionosphere. In summary, we have documented the first gigantic jets over the Indian mainland in the Indo-Gangatic plains. The jets reported here are the first observations from a thunderstorm developed purely over the land mass in Indo-Gangetic planes. Jets are of the negative polarity, transporting negative charge to the ionosphere consistent with past observations. Assuming 70 km distance from the clouds to the lower ionosphere, the charge moment suggests that 17–23 C charge is exchanged with the lower ionosphere. We also found that the Gigantic Jets were initiated close to the highest altitudes (~17 km) at the tropical tropopause boundary to the stratosphere. There are some unique features associated with the reported jets never documented in any previous report. The jets developed faster than usually observed, reaching up to ~37 km altitude in less than 5 ms, and they appeared also to be extinguished quickly, within ~40–80 ms. The GJ3 event appears markedly crooked, bending ~10 km towards the horizontal direction at ~50 Km altitude before branching upwards towards the ionosphere. We have also found that a bended jet might originate from an uncommon charge distribution vertically misaligned in the cloud. The observations underline that while thunderstorm clouds only rarely penetrate the tropopause, the electric influence reaches well into the stratosphere, both in discharges like TLEs and by the electrostatic field, electric currents and screening layers at cloud tops. Hence systematic, high-speed imaging of jets above strong convective systems during the monsoon season will give new knowledge of the elusive leader-streamer interaction fundamental to lightning propagation, because of the longer time- and spatial scales of the dilute, upper atmosphere.

## Methods

### Gigantic Jet Images analysis

The GJ images shown in Fig. 1 are from the site of Allahabad (25.4°N, 81.9°E) recorded by a video camera using a WATEC 902H2 Ultimate low light CCD (charge coupled device) camera coupled to a trigger software that captures optical event images and time-stamps the images using GPS receiver with accuracy of ~1 ms. In the analysis GLD360, lightning data from Vaisala Inc have been used to estimate the location and altitude of GJs.

### Magnetic measurements analysis

The magnetic field recordings shown in top panels in Fig. 3 were obtained from the Hylaty station in Poland (49.20°N, 22.54°E) and Duke University in the USA (35.97°N, 79.10°W). Both stations perform continuous measurements of the magnetic field component of electromagnetic waves in the frequency range 0.03 to 300 Hz (Hylaty) and 0.01 to 400 Hz (Duke) with the sampling frequency of 887.8 Hz and 2.5 kHz, respectively. Two orthogonal magnetic antennas are used at each site, which enable us to determine the direction of arrival of the recorded signal. Combining the two measurements we can infer the location of the source. Based on the magnetic field recordings we reconstructed the current moment waveform and charge moment change of the GJs. They were determined independently at each measurement site using their own techniques. In the case of Hylaty, we used an analytical radio wave propagation model and an inverse method to reconstruct the source parameters^24^. In the case of Duke, we used a Finite-difference time-domain numerical model of the Earth-ionosphere waveguide and performed forward simulations^31^. The obtained current moment and charge moment waveforms are aligned with the source time.

### Data Availability

The GJs and ELF magnetic field recordings data that support the findings of this study are available from authors RS, JM and SAC, but restrictions apply to the availability of these data as they are proprietary of respective institutions, and so are not publicly available. However data are available upon reasonable request from the authors for scientific and research purposes. Other data used are available in public domain.
